# Effect of obesity on oxygen uptake and cardiovascular dynamics during whole‐body and leg exercise in adult males and females

**DOI:** 10.14814/phy2.13705

**Published:** 2018-05-13

**Authors:** Simon Green, Eamon O'Connor, Catherine Kiely, Donal O'Shea, Mikel Egaña

**Affiliations:** ^1^ School of Science and Health Western Sydney University Sydney Australia; ^2^ School of Medicine Department of Physiology Trinity College Dublin Dublin Ireland; ^3^ Endocrinology St. Columcille's and St. Vincent's Hospitals Dublin Ireland

**Keywords:** Muscle vasodilation, O_2_ uptake, obesity, submaximal exercise, time constant

## Abstract

Obesity has been associated with a slowing of $\dot{V}$O_2_ dynamics in children and adolescents, but this problem has not been studied in adults. Cardiovascular mechanisms underlying this effect are not clear. In this study, 48 adults (18 males, 30 females) grouped according to body mass index (BMI) (lean < 25 kg·m^−2^, overweight = 25–29.9 kg·m^−2^, obese ≥30 kg·m^−2^) provided a fasting blood sample, completed a maximal graded exercise test and six bouts of submaximal exercise on a cycle ergometer, and performed two protocols of calf exercise. Dynamic response characteristics of $\dot{V}$O_2_ and leg vascular conductance (LVC) were assessed during cycling (80% ventilatory threshold) and calf exercise (30% MVC), respectively. Dynamic responses of cardiac output, mean arterial pressure and total systemic vascular conductance were also assessed during cycling based on measurements at 30 and 240 sec. The time constant of the second phase of the $\dot{V}$O_2_ response was significantly greater in obese than lean subjects (39.4 (9.2) vs. 29.1 (7.6) sec); whereas dynamic responses of cardiac output and systemic vascular conductance were not affected by BMI. For calf exercise, the time constant of the second growth phase of LVC was slowed significantly in obese subjects (22.1 (12.7) sec) compared with lean and overweight subjects (11.6 (4.5) sec and 13.4 (6.7) sec). These data show that obesity slows dynamic responses of $\dot{V}$O_2_ during cycling and the slower phase of vasodilation in contracting muscles of male and female adults.

## Introduction

Obesity, represented by a body mass index (BMI) greater than 30 kg·m^−2^ (Ortega et al. 2016), contributes to a loss of cardiorespiratory fitness. This is based largely on evidence of inverse associations between BMI, or other estimates of adiposity, and $\dot{V}$O_2peak_ (normalized to body mass) and performance during weight‐bearing tests (Wei et al. 2000; Drinkard et al. 2001; Tsiros et al. 2016). However, interpretation of these associations is complicated by the fact that, when comparisons are made between individuals of greatly different body masses, these peak exercise measurements are inherently correlated with body mass (i.e., $\dot{V}$O_2peak_) or directly influenced by body mass when exercise is weight‐bearing, such as treadmill and walk tests (Green et al. 2007; Krachler et al. 2015). Consequently, the physiological effects of obesity, independent of body mass per se, on cardiorespiratory function and $\dot{V}$O_2_ during exercise are less clear.

In contrast to peak exercise measurements, the dynamic response of $\dot{V}$O_2_ during submaximal exercise is less influenced by weight and provides unique insight into cardiorespiratory fitness linked to adjustments in levels of physical effort (Hughson et al. 2001). At the onset of moderate submaximal exercise, the $\dot{V}$O_2_ measured ‘at the lungs’ increases in a biphasic manner (Barstow and Mole 1991). The initial phase of this response is rapid, lasting for 15–40 sec, and is related to the abrupt increase in pulmonary blood flow (cardiac output) and hyperemia in contracting muscles (Whipp et al. 2002). The second phase evolves more slowly, exponentially, and it represents the O_2_ consumption by contracting muscles (Grassi et al. 1996). The time constant of this second (*τ*_2_) phase provides an estimate of the rate of change of this phase and perhaps best represents the speed of the dynamic response of $\dot{V}$O_2_ and underlying physiological processes linked to the supply and utilization of O_2_. In addition, this parameter is theoretically independent of its amplitude and, thus, an individual's body mass.

Evidence from human studies pertaining to the effects of obesity on *τ*_2_ are limited to studies of girls and boys (Lambrick et al. 2013) and male adolescents (Salvadego et al. 2010). This evidence suggests that a BMI > 30 kg·m^−2^ is associated with an increase in *τ*_2_ and slowing of the dynamic response of $\dot{V}$O_2_ by ~20–25%. As yet, the effects of BMI on *τ*_2_ or other aspects of $\dot{V}$O_2_ dynamics in healthy adults have not been established.

According to the Fick principle applied to $\dot{V}$O_2_ ($\dot{V}$O_2_ = $\dot{Q}$ × *a*‐*v*O_2_, where $\dot{Q}$ is blood flow, *a*‐*v*O_2_ is the arterial‐venous difference in blood O_2_ content), any effect of BMI on $\dot{V}$O_2_ dynamics measured at the lungs might be attributed to effects on dynamic responses of cardiac output (i.e., pulmonary arterial blood flow) and/or the arterial‐venous difference for O_2_ measured across the pulmonary circuit. Measurements of these systemic responses might shed light on whether BMI‐related changes in $\dot{V}$O_2_ dynamics are related to systemic rates of O_2_ delivery and/or O_2_ utilization. However, as we observed in patients with type 2 diabetes (MacAnaney et al. 2011; O'Connor et al. 2012; Kiely et al. 2014), it is also possible that a slowing in $\dot{V}$O_2_ dynamics is not explained by such systemic measurements but are associated with impaired responses of vasodilation measured in contracting muscles.

Therefore, in the this study, we tested the hypothesis that an increase in BMI is associated with a slowing of $\dot{V}$O_2_ dynamics (i.e., increase in *τ*_2_) during moderate cycling exercise in apparently healthy men and women. These subjects were not involved in regular physical activity and represented a ‘middle‐aged’ cohort. To explore some of the physiological factors underlying $\dot{V}$O_2_ dynamics, we measured cardiac output during cycling exercise and also the dynamic response characteristics of muscle vasodilation during single‐limb exercise with better temporal resolution than the cardiovascular measurements during cycling.

## Methods

### Subjects

Forty eight subjects (18 males, 30 females) participated in this study. Subjects were recruited from the general community of Dublin on the basis of the following inclusion criteria: aged 30*–*70 years; sedentary, ≤1 h of exercise per week and/or had not participated in a continuous exercise program for the previous 6 months; fasting glucose <6.1 mmol·L^−1^ and HbA_1c_ <6.5%; nonsmoker; systolic and diastolic blood pressures below 170 and 95 mmHg, respectively; and free of cardiovascular disease and other chronic diseases (neurological, respiratory, orthopedic) as assessed by a physician and medical health history questionnaire. Fifteen participants (10 females, 5 males) included in the study were taking 1*–*3 medications for the treatment of hypertension (*n = *3), hypercholesterolemia (*n* = 5), hormone replacement therapy (*n* = 2), hypothyroidism (*n* = 1), gastrointestinal complaints (*n* = 4), osteoporosis (*n* = 1), urinary incontinence (*n* = 1) and/or menstrual pain (*n* = 1). Individuals taking beta blockers were excluded from participation. Participants provided written informed consent prior to participation. All studies were approved by the Faculty of Health Science Research Ethics Committee (Trinity College Dublin) and conducted in accordance with the Declaration of Helsinki (2008).

### Baseline characteristics and BMI

Prior to exercise testing, each participant was assessed for BMI and waist‐to‐hip ratio, provided a fasting blood sample, and had their physical activity levels monitored for 5 days using triaxial accelerometry (StayHealthy, Monrovia, CA) (Rowlands et al. 2004). Ankle‐brachial blood pressure index (ABI) was also measured in both legs and all subjects had an ABI > 0.9, indicative of an absence of peripheral arterial disease. Baseline characteristics for females and males obtained using these techniques are shown in Table 1. For analyses involving BMI as a main effect (see Statistical analyses), participants were stratified into three BMI groups and classified as normal (BMI = 20–24.9 kg·m^−2^), overweight (BMI = 25–29.9 kg·m^−2^) and obese (BMI ≥ 30 kg·m^−2^).

**Table 1:** Baseline physical characteristics of subjects grouped by BMI category

	Lean	Overweight	Obese
Male/Female	6/10	6/10	6/10
Age (year)	51.6 (11.0)	56.4 (8.6)	54.0 (9.2)
Height (m)	1.69 (0.10)	1.67 (0.13)	1.68 (0.11)
Mass (kg)	66.8 (10.5)	76.5 (12.5)^a^	91.0 (11.9)^a^^,^ b
BMI (kg·m^−2^)	23.3 (1.4)	27.4 (1.3)^a^	32.0 (2.0)^a^^,^ b
Waist–hip ratio	0.90 (0.07)	0.96 (0.08)^a^	0.95 (0.04)^a^
Glucose (mmol·L^−1^)	4.4 (0.7)	4.8 (0.6)	4.9 (0.6)
HbA_1_c (%)	5.3 (0.2)	5.6 (0.3)	5.5 (0.5)
Cholesterol (mmol·L^−1^)	5.4 (1.1)	5.6 (1.4)	4.8 (0.7)
LDL‐C (mmol·L^−1^)	3.5 (0.8)	3.8 (1.2)	3.2 (0.5)
HDL‐C (mmol·L^−1^)	1.8 (0.4)	1.6 (0.5)	1.3 (0.4)
Triglycerides (mmol·L^−1^)	0.9 (0.4)	1.5 (0.7)	1.5 (0.6)
Systolic blood pressure (mmHg)	124 (8)	125 (11)	128 (19)
Diastolic blood pressure (mmHg)	79 (7)	81 (7)	87 (10)
ABI	1.10 (0.14)	1.15 (0.08)	1.11 (0.08)
Inactivity (h·day^−1^)	17.5 (1.9)	16.5 (1.5)	17.5 (2.1)
Light activity (h·day^−1^)	5.0 (1.4)	6.1 (1.3)	5.2 (1.5)
Moderate (h·day^−1^)	1.1 (0.5)	1.1 (0.5)	1.0 (0.6)
Vigorous (h·day^−1^)	0.5 (0.4)	0.4 (0.3)	0.3 (0.2)

Data are means (SD). BMI, body mass index; HbA1c, glycosylated hemoglobin; HDL, high‐density lipoprotein; LDL, low‐density lipoprotein; ABI, ankle‐brachial blood pressure index.

a Different from lean group (*P* ≤ 0.05).

b Different from overweight group (*P* ≤ 0.05).

### Exercise testing

Each subject completed three exercise testing sessions on separate days, at the same time of day, and with at least 72 h between sessions. During the first session subjects were familiarized with calf exercise and had their maximum voluntary contraction force assessed. During the second session subjects performed constant‐load and incremental calf exercise tests, and after 30 min of rest subjects then performed a graded exercise test on a cycle ergometer. During the third session subjects performed six constant‐load tests for measurement of $\dot{V}$O_2_ and cardiovascular dynamics. Subjects refrained from consuming caffeine and alcohol in the 24 h prior to testing and limited activity to normal activities of daily living. For premenopausal women (*n* = 9), testing occurred during the mid‐follicular phase of their menstrual cycle (days 5–12).

### Cycling: maximum graded exercise

Exercise testing was performed on an electrically braked cycle ergometer (Excalibur Sport, Lode, Groningen, Netherlands) at a cadence of 60 rpm. Subjects first completed a graded exercise test (GXT) to failure to determine their ventilatory threshold (*T*_vent_), $\dot{V}$O_2peak_ and peak heart rate. The GXT protocol consisted of a 3 min rest period, an initial power output of 40 W for 3 min, and then stepwise increases in power of 20 W (females) or 30 W (males) each 3 min until task failure (Egaña et al. 2007). *T*_vent_ was determined using the V‐slope method and $\dot{V}$O_2peak_ was the highest mean $\dot{V}$O_2_ recorded from consecutive 30 sec intervals during the test (Green and Askew 2018). We acknowledge that the use of the V‐slope method applied to incremental tests might overestimate *T*_vent_ in some subjects and contribute to the appearance of a slow phase in the dynamic response during ‘moderate’ exercise (see below). Gas exchange variables were measured breath‐by‐breath (Innocor, Innovision A/S, Odense, Denmark). Heart rate was recorded using a HR monitor (S610i, Polar Electro Oy, Finland) at 5 sec intervals.

### Cycling: $\dot{V}$O_2_ and cardiovascular dynamics during moderate exercise

Subjects performed 6, 9‐min bouts of cycling at 80% *T*_vent_, with each bout separated by 12 min of rest. These rest periods were sufficient to allow heart rate (*n* = 48) and blood lactate (*n* = 21) to return to baseline levels. Exercise was performed initially at 10 W (‘unloaded’ cycling) for 3 min before completing the remaining 6 min at 80% *T*_vent_. $\dot{V}$O_2_ and HR were recorded during the first four bouts, and cardiac output and arterial blood pressure were measured during the last two bouts. Evidence from others showed that dynamic response characteristics of $\dot{V}$O_2_ during moderate exercise were similar when estimated from repeated trials performed on the same day compared with separate days (Spencer et al. 2011).

$\dot{V}$O_2_ dynamics were assessed during the first four exercise bouts. $\dot{V}$O_2_ was measured breath‐by‐breath and responses from all four bouts were linearly interpolated to 1 sec intervals, time aligned, averaged (mean) and then smoothed using a 5 sec moving average filter (Keir et al. 2014). This averaged and smoothed response for each participant was fitted to a biexponential or triexponential function as follows:

(1) $$\dot{V}O_{2}\left(t\right)=\text{a +}\;A_{1}\left(1-e^{-\left(t-{\mathrm{TD}}_{1}\right)/\tau_{1}}\right)F_{1}+A_{2}\left(1-e^{-\left(t-{\mathrm{TD}}_{2}\right)/\tau_{2}}\right)F_{2}$$

(2) $$\dot{V}O_{2}\left(t\right)=\text{a +}\;A_{1}\left(1-e^{-\left(t-{\mathrm{TD}}_{1}\right)/\tau_{1}}\right)F_{1}+A_{2}\left(1-e^{-\left(t-{\mathrm{TD}}_{2}\right)/\tau_{2}}\right)F_{2}+A_{3}\left(1-e^{-\left(t-{\mathrm{TD}}_{3}\right)/\tau_{3}}\right)F_{3}$$

where parameter *a* represents $\dot{V}$O_2_ during unloaded exercise, A_1_‐A_3_ are phase amplitudes, TD_1_‐TD_3_ are phase delays, and *τ*_1_‐τ_3_ are time constants of each phase. The conditional expressions (F1 and F2) limit the fitting of a particular phase to the period at and beyond the time delay associated with that phase. Parameter estimates of the best‐fit function (Reeder and Green 2012) were used and only estimates representing the first two phases are presented. The presence of a third phase was detected in 9 participants, but the amplitude of this phase was small (mean (SD) = 98 (48) mL^·^min^−1^), it was detected in members of each group (lean = 4; overweight = 4; obese = 1), and its presence does not appear to significantly affect the parameter estimates of the earlier phases (Wilkerson et al. 2004). $\dot{V}$O_2_ responses were fitted to both functions using a two‐step fitting process where data lying outside the 95% prediction interval during the initial fit were excluded and the fitting was performed a second time. Fitting was performed using the Levenburg–Marquardt algorithm and a weighted least‐squares nonlinear regression procedure (TableCurve 2D, Systat).

The $\dot{V}$O_2_ at the end of moderate exercise (EndA) was calculated as follows:

(3) $$\text{EndA}=\text{a +}\;A_{1}\left(1-e^{-(360-{\mathrm{TD}}_{1}/\tau 1)}\right)+A_{2}\left(1-e^{-(360-{\mathrm{TD}}_{2}/\tau_{2})}\right)$$

The $\dot{V}$O_2_ gain (mL^·^min^−1·^W^−1^) was calculated as the difference between EndA and $\dot{V}$O_2_ during unloaded cycling (parameter *a*) normalized to the difference in power outputs between moderate exercise and unloaded cycling.

The dynamic response of heart rate during the above‐mentioned exercise bouts was also estimated. Four time‐series of heart rate responses measured at 5 sec intervals were averaged to yield a single time‐series of heart rate data for each subject and fitted to the monoexponential function,

(4) $$\text{HR}\left(t\right)=a+A\left(1-e^{-(t-\mathrm{TD})/\tau }\right)$$

where parameter *a* is a baseline heart rate estimated for the 3 min of unloaded cycling (10 W), A is the amplitude of the heart rate response during exercise at 80% *T*_vent_, TD is the delay in rise of heart rate after exercise onset, and *τ* is the time constant of the response. Fitting procedures were identical to that described for $\dot{V}$O_2_.

Cardiac output dynamics were assessed during the fifth and six exercise bouts. Cardiac output (CO, L·min^−1^) was measured using inert gases (sulfur hexafluoride and nitrous oxide) rebreathing technique (Innocor, Innovision A/S, Odense, Denmark) at rest and at 30 sec and 240 sec of exercise. Rebreathing maneuvers lasted ~10 sec. Heart rate was recorded every 5 sec (S610i, Polar Electro Oy, Finland) and along with simultaneous CO measurements used to estimate stroke volume (SV = CO/HR, mL·beat^−1^). Measurements of systolic and diastolic blood pressure (mmHg) were made at the same times as CO measurements using manual sphygmomanometry and used to estimate mean arterial pressure (MAP = SBP/3 + 2^.^DBP/3; mmHg). Systemic vascular conductance (SVC) was calculated, using these measurements (SVC = CO/MAP, mL·min^−1^·mmHg^−1^). Final values for all cardiovascular variables were averaged from responses during the two submaximal exercise bouts.

### Calf exercise and haemodynamics

Calf exercise involved plantar flexors of the right leg and was performed in the supine position on a custom‐built ergometer (Egaña and Green 2005). For both protocols contractions were static and performed intermittently. Subjects were secured to the ergometer with a harness that minimized the backward displacement of the body during each contraction. Feet were attached to an immobile footplate, connected to a load cell, against which force was applied as subjects attempted to plantar‐flex the foot. The measured force was amplified and sampled at 40 Hz before being processed by a PowerLab analog‐to‐digital converter (ML 795, AD Instruments). Force was displayed on a screen visible to the subject (Chart v6.0, AD Instruments) to help them control their effort and conform to specific instructions given by the investigator.

On one day, subjects were familiarized with performing intermittent contractions, practiced the incremental and constant‐load protocols, and then performed a series of maximal voluntary contractions to determine the maximum voluntary contraction force (MVC). On a subsequent day, subjects performed three bouts of intermittent contractions (2 sec contraction, 4 sec relaxation) at 30%MVC with 10 min rest periods in between bouts (Egaña and Green 2005; Kiely et al. 2014). This intensity corresponds to approximately 50% of the peak force achieved during an incremental protocol, does not evoke a third growth phase observed at higher forces (Reeder and Green 2012), and it can be sustained with a minimal rate of fatigue over 30 min (Egaña and Green 2007). On this basis the exercise intensity might be considered to be mild‐to‐moderate and somewhat consistent with the ‘moderate’ intensity of cycling exercise used in this study. Subjects then performed an incremental test to failure (intermittent contractions as above), beginning with contractions at a force of 100 N for 2 min and then stepwise increases in force each 2 min of 200 N for males and 150 N for females until task failure (Kiely et al. 2014). Peak force (*F*_peak_) achieved during this test was defined as the highest force that a subject could sustain for at least 60 sec.

At rest and during calf exercise, leg blood flow (LBF) and MAP were measured simultaneously and used to calculate leg vascular conductance (LVC = LBF/MAP). LBF was assessed using venous occlusion plethysmography (Egaña and Green 2005). When compared with Doppler ultrasound, this technique provides similar estimates of limb blood flow and vascular conductance during incremental and constant‐load calf exercise protocols (Green et al. 2011; Murphy et al. 2018), as well as similar parameter estimates used to define the dynamic response of LVC (Murphy et al. 2018). To measure LBF, a mercury‐silastic strain gauge (Hokanson EC‐6) was placed around the widest part of the subject's right calf and connected to the plethysmograph (Hokanson EC‐6). A cuff (Hokanson) placed around the upper right thigh of the subject was inflated to a constant pressure of ~50 mmHg for the duration of the exercise. The pressure of ~50 mmHg was chosen to occlude venous return without interfering with arterial flow into the leg. LBF during exercise was assessed by measuring the change in leg volume detected by the strain gauge over the 4 sec relaxation period between contractions. Beat‐to‐beat heart rate (HR) and systolic & diastolic blood pressures were measured at rest and during exercise using either applanation tonometry of the radial artery (COLIN CBM7000, Japan) or the volume clamp method at the level of the finger (Finometer, Finapres Medical Systems B.V., the Netherlands). A pilot reliability study was performed (*n* = 10) to ensure that the readings attained for each of these two methods were accurate when recordings during an incremental calf plantar‐flexion exercise were taken using both units simultaneously (intra‐correlation coefficient = 0.82). MAP was calculated from systolic and diastolic pressures (MAP: 0.33 systolic BP + 0.66 diastolic BP). The plethysmographic estimates of LBF, expressed relative to the resting limb volume (mL·100 mL^−1^·min^−1^), were converted to millilitres per minute (mL·min^−1^) using an estimate of each subject's leg volume obtained from anthropometric measurements of the leg (Clarys and Marfell‐Jones 1986a). LVC was calculated as the ratio of LBF to MAP (LVC = LBF/MAP, mL·min^−1^·mmHg^−1^). Leg muscle mass was estimated using an anthropometrical approach (Clarys and Marfell‐Jones 1986b).

LVC responses (mL·min^−1^·mmHg^−1^) for the three exercise bouts were averaged to yield a temporal profile of LVC for each subject. In accordance with our recent description of the structure of this dynamic response in young healthy subjects (Reeder and Green 2012; Murphy et al. 2018), as well as its verification in older healthy subjects and overweight and obese subjects with type 2 diabetes (Kiely et al. 2014), these averaged, individual responses were fitted to the following function,

(5) $$\text{LVC}\left(t\right)=A_{0}+A_{1}\left(1-e^{-\left(t-{\mathrm{TD}}_{1}\right)/\tau_{1}}\right)F_{1}-A_{2}\left(1-e^{-(t-{\mathrm{TD}}_{2})/\tau_{2}}\right)F_{2}+A_{3}\left(1-e^{-\left(t-{\mathrm{TD}}_{3}\right)/\tau_{3}}\right)F_{3}-A_{4}\left(1-e^{-\left(t-{\mathrm{TD}}_{4}\right)/\tau_{4}}\right)F_{4}$$

where A_0_ is the baseline response immediately prior to the initial contraction, A_1_–A_4_ are amplitudes, TD_1_–TD_4_ are time delays, and *τ*_1_–*τ*_4_ are time constants of the first (rapid growth), second (rapid decay), third (slow growth), and fourth (slow decay) phases, respectively. The parameters F1–F4 are conditional expressions that limit the fitting of a particular phase to the period at and beyond the time delay associated with that phase. LVC data were fitted using a weighted least‐squares nonlinear regression procedure and the Marquardt–Levenberg algorithm (TableCurve 2D, Jandel Scientific). For all models, data that exceeded the 95% prediction intervals during an initial fit of a model were excluded. No more than four data points were removed from the original time‐series of data. Some subjects did not display a second decay phase and so only parameters describing the first three phases were analyzed.

The steady‐state amplitude of the LVC response, referred to as End A, was calculated as follows:

(6) $$\begin{array}{cc} \text{End A} & =\text{a +}\;A_{1}\left(1-e^{-(360-{\mathrm{TD}}_{1}/\tau 1)}\right)\\ & -A_{2}\left(1-e^{-(360-{\mathrm{TD}}_{2}/\tau_{2})}\right)+A_{3}\left(1-e^{-(360-{\mathrm{TD}}_{3}/\tau_{3})}\right)\\ & +A_{4}\left(1-e^{-(360-{\mathrm{TD}}_{4}/\tau_{4})}\right) \end{array}$$

### Statistical analysis

Data were normally distributed and so effects of BMI (3 levels) were initially tested using one‐way ANOVA. A two‐way ANOVA was used to explore interactions involving BMI and sex and verify main effects of BMI. The level of significance was set at *P *≤* *0.05. All values are expressed as mean and SD. All statistical analyses were performed using SPSS v.24 (IBM SPSS).

## Results

### BMI

Data for physical characteristics are shown in Table 1. Differences in BMI between lean, overweight and obese groups (*F*_2,45_ = 123, *P *<* *0.05) were a function of differences in body mass (*F*_2,45_ = 17.1, *P *<* *0.05). Other physical characteristics, blood‐borne measurements and physical activity levels were similar between groups. Calf skinfold measurements, required for estimation of leg muscle mass, were significantly lower in lean (11.3 (4.1) mm) compared with obese (14.5 (4.2) mm) subjects and intermediate values were observed for overweight subjects (12.4 (4.8) mm).

Peak responses during graded exercise are shown in Table 2. $\dot{V}$O_2peak_ normalized to body mass varied as a function of BMI (*F*_2,45_ = 5.6, *P *<* *0.05) and was significantly different between the three groups. No other peak responses were significantly affected by BMI.

**Table 2:** Peak physiological responses measured during maximal graded exercise grouped by BMI category

	Lean (*n* = 16)	Overweight (*n* = 16)	Obese (*n* = 16)
Male/Female	6/10	6/10	6/10
Workrate_peak_ (W)	149 (48)	146 (43)	143 (50)
$\dot{V}$O_2peak_ (L·O_2_·min^−1^)	2.12 (0.88)	2.03 (0.70)	2.00 (0.76)
$\dot{V}$O_2peak_ (mL·O_2_·kg^−1^·min^−1^)	30.5 (9.0)	26.0 (5.6)	21.6 (6.2)^a^,^b^
$\dot{V}$_Epeak_ (L·min^−1^)	74.9 (23.9)	72.0 (18.0)	71.0 (28.3)
HR_peak_ (beats·min^−1^)	164 (15)	159 (17)	158 (11)
RER_peak_	1.10 (0.05)	1.11 (0.05)	1.10 (0.05)
*T*_vent_ (W)	122 (45)	123 (34)	120 (40)
*T*_vent_ (L·O_2_·min^−1^)	1.55 (0.69)	1.49 (0.59)	1.47 (0.62)
*T*_vent_ (%$\dot{V}$O_2peak_)	73.9 (8.7)	72.7 (10.9)	71.8 (8.7)

Data are means (SD). $\dot{V}$O_2_, peak oxygen consumption; $\dot{V}$_Epeak_, peak minute ventilation; HR_peak_, peak heart rate; RER_peak_, peak respiratory exchange ratio; *T*_vent_, ventilatory threshold.

a Different from lean group (*P *≤* *0.05).

b Different from overweight group (*P *≤* *0.05).

**Figure 1:**
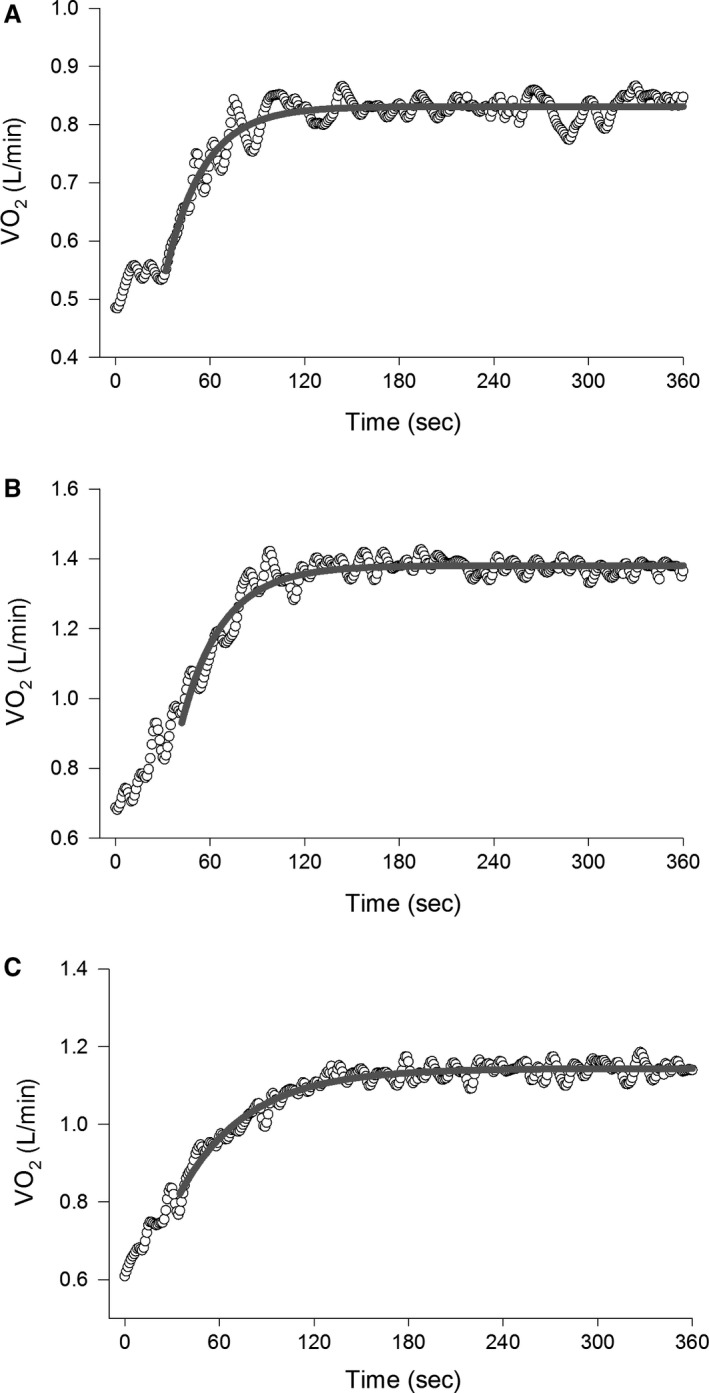
Representative responses of $\dot{V}$O_2_ dynamics during moderate exercise (80% *T*_vent_) with lines of best fit of the second phase related to empirical modeling (Eq. (1)) for a lean (A), overweight (B) and obese (C) subject. Note the relatively slower response of the second phase of the $\dot{V}$O_2_ response in the obese subject.

Parameters describing the dynamic response of $\dot{V}$O_2_ during cycling at 80% *T*_vent_ are shown in Table 3. Two obese subjects failed to complete the required exercise bouts and so the following analyses are based on 46 subjects. BMI had a significant effect on *τ*_2_ (*F*_2,43_ = 5.3, *P *<* *0.05), which was significantly greater in obese than lean groups. By contrast, BMI did not significantly affect any other parameter or the gain of $\dot{V}$O_2_. Temporal responses of $\dot{V}$O_2_ from representative lean, overweight and obese individuals are shown in Figure 1.

**Table 3:** Parameter estimates related to $\dot{V}$O_2_ dynamics grouped by BMI category

	Lean (*n* = 16)	Overweight(*n* = 16)	Obese (*n* = 14)
Male/Female	6/10	6/10	6/8
Workrate (W)	98 (36)	99 (27)	97 (33)
*a* (L·min^−1^)	0.58 (0.14)	0.63 (0.13)	0.64 (0.16)
A_1_ (L·min^−1^)	0.35 (0.22)	0.39 (0.14)	0.31 (0.17)
TD_1_ (sec)	4.7 (4.0)	5.5 (4.5)	4.4 (3.4)
*τ*_1_(sec)	13.0 (6.9)	14.7 (5.7)	8.9 (5.6)
A_2_ (L·min^−1^)	0.62 (0.35)	0.57 (0.24)	0.65 (0.31)
TD_2_ (sec)	32.3 (8.2)	31.0 (6.9)	27.8 (8.9)
*τ*_2_ (sec)	29.1 (7.6)	34.7 (9.0)	39.4 (9.2) a
End A (L·min^−1^)	1.55 (0.54)	1.59 (0.40)	1.60 (0.51)
$\dot{V}$O_2_ Gain (mL·min^−1^·W^−1^)	10.9 (1.7)	10.8 (0.8)	11.1 (1.3)

Data are means (SD). *a,* amplitude during unloaded cycling; A_1_ and A_2_ amplitudes, TD_1_ and TD_2_, time delays and *τ*_1_ and *τ*_2_, time constants of the first and second phases, respectively.

a Different from lean group (*P *≤* *0.05).

**Figure 2:**
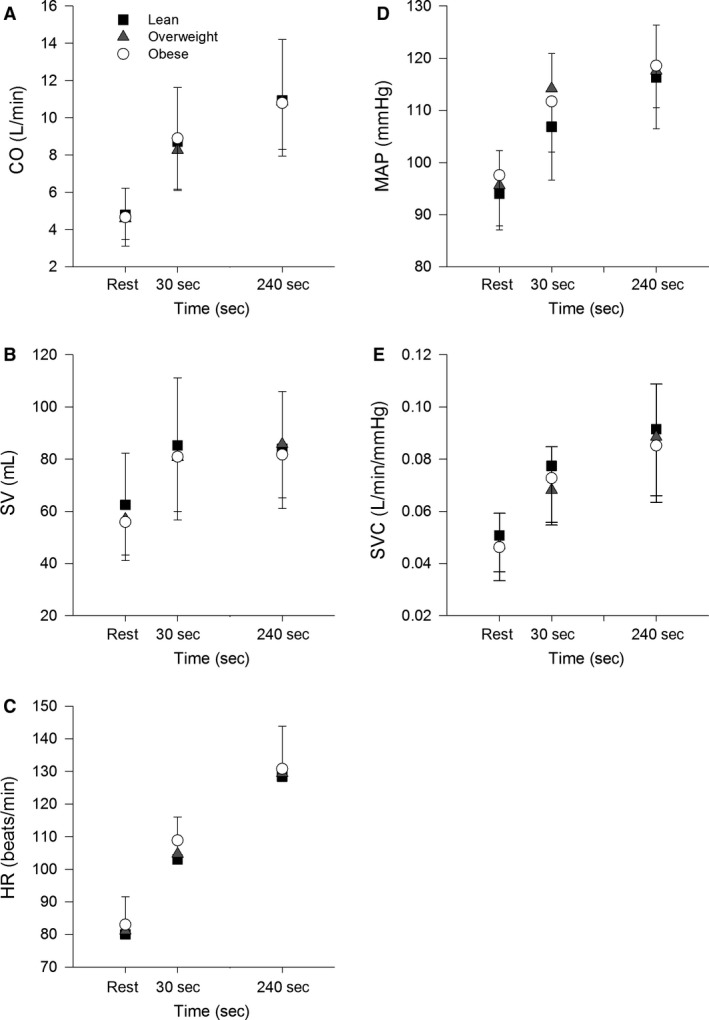
Mean responses of cardiac output (CO) (A), stroke volume (SV) (B), heart rate (HR) (C), mean arterial pressure (MAP) (D) and systemic vascular conductance (SVC) (E) at rest and during moderate exercise (80%*T*_vent_) at 30 and 240 sec in lean (square symbol), overweight (circle symbol) and obese (triangle symbol) subjects.

Cardiovascular responses during cycling at 80% *T*_vent_ are shown in Figure 2. Again, two obese subjects failed to complete this testing and so analyses of cardiac output, heart rate and stroke volume are based on 46 rather than 48 subjects. In addition, successful blood pressure recordings and estimates of MAP and SVC were only obtained in 36 subjects (12 lean, 10 overweight, 14 obese). None of the responses measured at rest, 30 sec and 240 sec were significantly different between groups. Ratios of the change of these responses over the initial 30 sec of exercise relative to responses at 240 sec are shown in Table 4. The ratio of change in MAP was significantly affected by BMI (*F*_2,33_ = 7.2, *P *<* *0.05) and significantly different between lean and overweight groups. None of the other ratios were significantly affected by BMI.

**Table 4:** Estimates of dynamic responses of cardiovascular variables based on changes from rest over the initial 30 and 240 sec of exercise expressed as ratios (Δ30/Δ240)

	Lean (*n* = 16)	Overweight (*n* = 16)	Obese (*n* = 14)
Male/Female	6/10	6/10	6/8
HR	0.29 (0.11)	0.30 (0.10)	0.34 (0.26)
SV	0.40 (0.32)	0.44 (0.31)	0.49 (0.34)
CO	0.62 (0.14)	0.58 (0.19)	0.66 (0.15)
MAP	0.56 (0.25)	0.86 (0.17)^a^	0.73 (0.20)
SVC	0.65 (0.14)	0.49 (0.29)	0.66 (0.15)

A higher ratio indicates a faster response. For example, the first estimate (mean = 0.29) indicates that ~30% of the change in heart rate over 240 sec of exercise was achieved in the first 30 sec of exercise. Data are means (SD). HR, heart rate; SV, stroke volume; CO, cardiac output; MAP, mean arterial pressure; SVC, systemic vascular conductance. Note: sample sizes for MAP and SVC were 12 (lean), 10 (overweight) and 14 (obese).

a Different from lean group (*P *≤* *0.05).

The time constant of the heart rate response was also not different between lean (60.9 (10.2) sec), overweight (57.1 (11.9) sec) and obese subjects (66.7 (21.6) sec). None of the other parameters describing the HR response (i.e., baseline, amplitude, time delay) were significantly different between the three groups.

Peak responses during incremental calf exercise, as well as baseline measurements of the leg, grouped by BMI are shown in Table 5. There were significant differences in leg muscle mass (*F*_2,43_ = 9.4, *P *<* *0.05) and leg volume (*F*_2,43_ = 4.1, *P *<* *0.05) between groups. By contrast, MVC and performance of the exercise task were not significantly affected by BMI. Peak LBF, LVC and MAP were also not significantly affected by BMI, although the effect on MAP was close to significance (*F*_2,41_ = 3.1, *P* = 0.06).

**Table 5:** Characteristics of leg anthropometry, strength, and responses to incremental calf exercise according to BMI category

	Lean (*n* = 16)	Overweight (*n* = 15)	Obese (*n* = 15)
Male/Female	6/10	6/9	6/9
Leg muscle mass (kg)	1.64 (0.31)	1.91 (0.28)^a^	2.12 (0.33)^a^
Leg volume (mL)	2437 (339)	2685 (363)	2833 (467)^a^
MVC (N)	737 (224)	805 (333)	819 (317)
MVC (N·kg^−1^ leg muscle mass)	443 (83)	410 (125)	379 (105)
Exercise time (sec)	416 (110)	434 (125)	440 (87)
Force_peak_(N)	525 (203)	567 (224)	557 (161)
Force_peak_ (%MVC)	71.0 (13.8)	70.8 (9.5)	71.6 (15.3)
LBF_peak_(mL·min^−1^)	644 (231)	677 (344)	716 (350)
MAP_peak_ (mmHg)	101 (14)	106 (12)	113 (14)
LVC_peak_ (mL·min^−1·^mmHg^−1^)	6.76 (2.93)	6.52 (3.27)	6.27 (3.03)

Data are means (SD). MVC, maximal voluntary contraction; LBFpeak, peak leg blood flow; MAPpeak, peak mean arterial pressure LVCpeak, peak leg vascular conductance.

a Different from lean group (*P *≤* *0.05).

**Figure 3:**
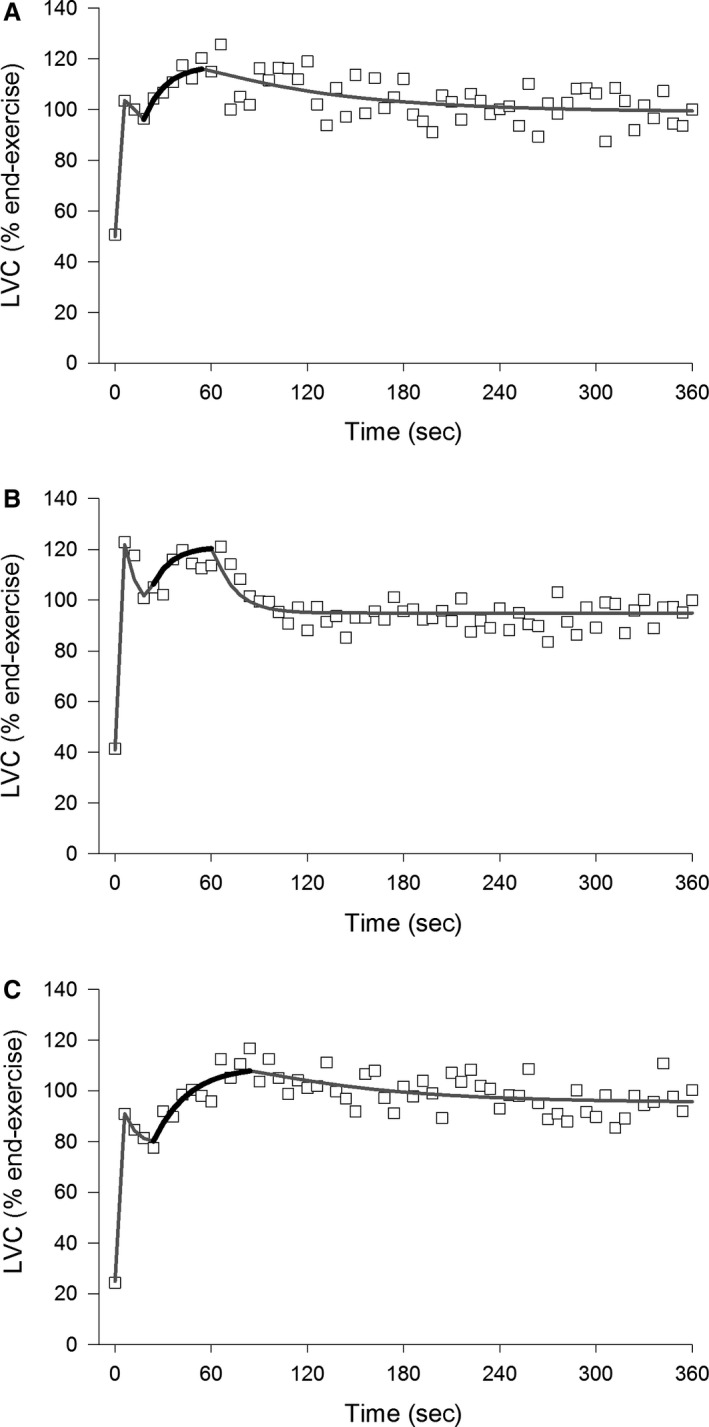
Representative responses of leg vascular conductance (LVC) dynamics during calf exercise (30%MVC) with lines of best fit related to empirical modeling (Eq. (5)) for a lean (A), overweight (B) and obese (C) subject. The dark‐lined segment of the line of best fit represents the third phase and illustrates the slower rise in the obese subject. LVC values are expressed relative to the end‐exercise amplitude to facilitate comparisons between participants.

LVC responses during submaximal calf exercise (30% MVC) are shown in Figure 3. For technical reasons, data from one overweight subject and one obese subject were not analyzed and so the following analyses are based on a sample of 46 subjects. Estimates of parameters describing the first three phases of this response are shown in Table 6. BMI had significant effects on the time delay of phase 2 (TD_2_: *F*_2,43_ = 4.5, *P *<* *0.05) and time constant of phase 3 (*τ*_3_: *F*_2,43_ = 6.5, *P *<* *0.05), with the latter being significantly greater in the obese group compared with the lean and overweight groups. There was also a more modest effect of BMI on TD_3_ which did not reach significance (*F*_2,43_ = 2.4, *P* = 0.10). None of the phase amplitudes or total amplitude were affected by BMI.

**Table 6:** Dynamic response characteristics of leg vascular conductance during calf exercise at 30%MVC according to BMI category

	Lean (*n* = 16)	Overweight (*n* = 15)	Obese (*n* = 15)
Male/Female	6/10	6/9	6/9
A_0_ (mL·min^−1^·mmHg^−1^)	0.63 (0.29)	0.86 (0.51)	0.60 (0.29)
A_1_ (mL·min^−1^·mmHg^−1^)	2.17 (1.23)	2.29 (2.07)	1.91 (1.15)
TD_1_ (sec)	1.2 (0.9)	1.3 (1.0)	0.8 (0.9)
*τ*_1_ (sec)	4.1 (1.7)	5.4 (4.1)	3.4 (3.0)
A_2_ (mL·min^−1^·mmHg^−1^)	0.93 (0.88)	0.67 (0.60)	0.47 (0.39)
TD_2_ (sec)	7.8 (5.1)	15.1 (9.8)^a^	10.2 (4.6)
*τ*_2_ (sec)	7.1 (3.5)	9.6 (5.3)	11.1 (11.3)
A_3_ (mL·min^−1^·mmHg^−1^)	1.13 (0.94)	0.96 (0.85)	1.10 (0.75)
TD_3_ (sec)	19.8 (8.5)	27.3 (11.4)	23.9 (8.5)
*τ*_3_ (sec)	11.6 (4.5)	13.4 (6.7)	22.1 (12.7)^a^,^b^
EndA (mL·min^−1^·mmHg^−1^)	3.45 (1.71)	3.47 (2.12)	2.98 (1.49)

Data are means (SD). A_0_ baseline amplitude; A_1_, A_2_ and A_3_, amplitudes, TD_1_, TD_2_ and TD_3_, time delays and *τ*_1_, *τ*_2_ and *τ*_3_, time constants of the first (rapid growth), second (rapid decay) and third (slow growth) phases, respectively.

a Different from lean group (*P *≤* *0.05).

b Different from overweight group (*P *≤* *0.05).

### BMI and sex

For variables identified in Tables 1, 2, 3, 4, 5, 6, two‐way ANOVA was performed to test for main and interactive effects involving sex and BMI. A large number of variables were significantly affected by sex (main effect), as shown in Table 7. By contrast, only two variables were associated with significant BMI‐by‐sex interactions and which related to timing of the onsets of the second phase (TD_2_) and third phase (TD_3_) of the LVC response. For both TD_2_ (sex‐by‐BMI: *F*_2,40_ = 6.0) and TD_3_ (sex‐by‐BMI: *F*_2,40_ = 5.4), a positive effect of BMI on this parameter was only observed in females and not males. By contrast, weak and nonsignificant interactions (*F*_2,40_ ≤ 1.3) were observed for the time constants of the three phases of the LVC response.

**Table 7:** Variables identified in Tables 1, 2, 3, 4, 5, 6 which were significantly different (ANOVA *P* < 0.05) between males (♂: *n* = 18) and females (♀: *n* = 28). Unless indicated, values for males were greater than values for females

Physical	GXT	$\dot{V}$O_2_ dynamics	Leg
Height	Workrate_peak_ (W)	*a*	Leg mass
Mass	$\dot{V}$O_2peak_ (mL·kg^−1^·min^−1^)	A_1_	Leg volume
FPG	$\dot{V}$O_2peak_ (L·min^−1^)	A_2_	MVC (N)
HbA_1_C		EndA	MVC (N·kg^−1^)
ABI	*V*_Epeak_ (L·min^−1^)		ExTime (sec)
DBP	*T*_vent_ (W)		*F*_peak_ (N)
HDL: (♂ < ♀)			MAP_peak_
			A_0_
			A_2_
			*τ*_1_ (♂ < ♀)

### Correlations with blood‐borne markers

Significant associations (*P *≤* *0.05) between baseline physical characteristics (Table 1), $\dot{V}$O_2_ *τ*_2_ and LVC*τ*_3_ were explored using the Pearson correlation coefficient (*n* = 44–46). Both $\dot{V}$O_2_ *τ*_2_ and LVC*τ*_3_ were significantly correlated with BMI (*r* = 0.35, 0.36) and the correlation between them was close to significance (*r* = 0.28, *P *=* *0.07). No other significant correlations were observed for $\dot{V}$O_2_ *τ*_2_. For LVC*τ*_3_, there were significant correlations with body mass (*r* = 0.40), HDL (*r* = −0.31) and triglycerides (*r* = 0.30), and the correlation with total cholesterol was close to significance (*r* = −0.28, *P* = 0.07).

## Discussion

This study tested the hypothesis that obesity, represented by a BMI > 30 kg·m^−2^, slows $\dot{V}$O_2_ dynamics during moderate cycling exercise in a cohort of middle‐aged and apparently healthy adults. The present findings support this hypothesis and show that the time constant of the second phase (*τ*_2_) was significantly greater in obese compared with lean subjects, with intermediate *τ*_2_ values for overweight subjects. By contrast, estimates of the rates of change in cardiac output and systemic vascular conductance were not significantly affected by BMI. For calf exercise, BMI was associated with a slowing of the second growth phase of the leg vascular conductance response (i.e., *τ*_3_), a phase which coincides with phase 2 of the $\dot{V}$O_2_ response. These findings show that BMI impairs $\dot{V}$O_2_ dynamics and vasodilation in contracting muscles of apparently healthy but sedentary adults.

### BMI and $\dot{V}$O_2_ dynamics during cycling

This study provides the first evidence that BMI affects $\dot{V}$O_2_ dynamics (*τ*_2_) in apparently healthy adults and that this effect was similar between males and females. This effect also appeared to be proportional to BMI across the three categories (Table 3) and was greatest for a level of obesity which can be considered to be mild (BMI ≈ 32 m^·^kg^−2^). The potency of this effect of mild obesity – an average of 35% increase in *τ*_2_‐when compared with lean subjects is similar to the effect of type 2 diabetes on *τ*_2_ in males (36%) and females (34%) averaged from several studies (Green et al. 2015). These observations are also similar to the effects of mild obesity on *τ*_2_ observed in girls and boys (Lambrick et al. 2013) and male adolescents (Salvadego et al. 2010) of ~20–25%. Collectively, these findings suggest that increases in body mass leading to obesity induce a considerable slowing of the dynamic response of $\dot{V}$O_2_ in inactive males and females and across a wide range of ages.

### Cardiovascular dynamics during cycling

Mechanisms underlying any effect of BMI on $\dot{V}$O_2_ dynamics must influence the rates of O_2_ delivery to and/or utilization by contracting muscles. There is evidence from animal studies that obesity impairs control of muscle vasodilation and perfusion (Frisbee 2003; Ribiero et al. 2005; Xiang et al. 2005, 2006; Frisbee et al. 2009), although how this relates to the present findings pertaining to exercising humans is not clear. The measurement in human subjects of the ‘dynamic’ responses of O_2_ delivery and utilization at a temporal resolution consistent with breath‐by‐breath gas analysis is a considerable challenge (Grassi et al. 1996). This is further complicated by the need to describe behaviors of Fick variables for $\dot{V}$O_2_ (blood flow, *a*‐*v*O_2_) measured both at the lungs and for a large number of contracting muscles. In this study, we used two approaches to shed light on aspects of cardiovascular dynamics at a systemic level during whole‐body exercise and on vascular dynamics during intermittent contractions in an isolated limb.

First, under the same exercise conditions used to assess $\dot{V}$O_2_ dynamics, we estimated the dynamic response of cardiac output based on the ratio of change in cardiac output during the early period of exercise (30 sec) relative to its change after 4 min (Table 4). Previously, we showed that this ratio and *τ*_2_ ($\dot{V}$O_2_) were increased by training in individuals with type 2 diabetes (MacAnaney et al. 2012), suggesting that this ratio was sensitive to changes in cardiac output and $\dot{V}$O_2_ dynamics. However, neither this estimate of the initial rate of change nor the absolute values of cardiac output during exercise were affected by BMI. In addition, BMI did not affect the dynamic responses of stroke volume and heart rate (Table 4) or estimates of heart rate dynamics obtained from empirical modelling (Eq. (4)). This evidence suggests that cardiac output dynamics during moderate exercise are not affected by obesity and do not contribute to the slowing of $\dot{V}$O_2_ dynamics with increasing BMI.

Large changes in cardiac output during exercise relative to much smaller changes in mean arterial pressure reflect an increase in systemic vascular conductance. During exercise at a moderate intensity, the increase in systemic vascular conductance is affected mainly by an increase in vascular conductance in contracting muscles (Mortensen et al. 2005). Thus, estimates of the dynamic response of systemic vascular conductance might provide insight into haemodynamic effects of BMI in contracting muscles. In this study, the average change in systemic vascular conductance from baseline to the fourth minute of moderate exercise was similar between lean (81%), overweight (90%) and obese (80%) subjects, consistent with the similar power outputs at which these groups exercised. Approximately 50–65% of this change in systemic vascular conductance was achieved during the first 30 sec of exercise and the ratio which reflects this estimate (Table 4) was also not different between these groups. This suggests that BMI does not affect the dynamic response of systemic vascular conductance during cycling and possibly the underlying response (vasodilation) in contracting muscles.

However, for several reasons this interpretation should be made with caution and direct measurements of muscle hyperemic responses during cycling are required to confirm it. Whether or not impaired hyperemic responses in contracting muscles are manifest in systemic measurements might also depend on the relative distribution of blood flows to different tissues and/or magnitude of a local hyperemic response relative to a larger systemic value. It is also tempting to use these systemic measurements to infer that dynamic responses of O_2_ utilization in contracting muscles, reflected in calculations of *a*‐*v*O_2_ from $\dot{V}$O_2_ and cardiac output measurements (Fick principle), are slowed by obesity given it affects $\dot{V}$O_2_ but not cardiac output dynamics. However, we are unable to verify this because any estimate of *a*‐*v*O_2_ based on present cardiac output and $\dot{V}$O_2_ measurements corresponds to the initial, ‘cardiodynamic’ phase which does not represent O_2_ uptake by contracting muscles (Grassi et al. 1996). This highlights a limitation of measuring cardiac output using an inert‐gas rebreathing technique restricted to a measurement frequency of 2 min or more and the challenge of optimizing the timing of the first exercise measurement.

### Vasodilation during isolated muscle contractions

Ideally, establishing the role of muscle blood flow and O_2_ supply in BMI‐related effects on $\dot{V}$O_2_ dynamics requires these variables to be measured during cycling. This, however, is not possible with available techniques and probably requires adaptation of a technique, such as Doppler ultrasound, with the required temporal resolution of measurement applied in the correct location (e.g., aortic‐iliac bifurcation) to ‘sample’ all primary muscles involved. We chose a lesser alternative but one which enabled an accurate description of the structure of the dynamic response of vasodilation in contracting muscles (Reeder and Green 2012), represented by the measurement of LVC.

Using a model of isolated limb (calf) exercise and intermittent contractions, we previously showed that the response of LVC in contracting muscles consisted of 3–4 phases which alternate between growth and decay in vascular conductance. This dynamic structure was observed in younger, healthy individuals (Reeder and Green 2012; Donnelly and Green 2013; Murphy et al. 2018), older healthy individuals (Reeder and Green 2011), as well as middle‐aged subjects with type 2 diabetes (Kiely et al. 2014). This same structure was observed in the present cohort and, because the fourth phase was not observed in all subjects, our analyses were restricted to the first three phases.

The first and third phases of the leg vascular conductance response are ‘growth’ phases which represent an initial and rapid vasodilation followed by a smaller and slower rate of vasodilation, respectively. The second of these growth phases begins at a time (time delay ≈ 20–30 sec) similar to the onset of phase 2 of the $\dot{V}$O_2_ response. In the present study, this second growth phase of LVC was significantly slower (higher *τ*_3_) in obese subjects compared with lean and overweight subjects. Although we did not analyze the dynamic response characteristics of leg (muscle) blood flow, with this experimental model the leg vascular conductance and blood flow responses are nearly identical (Reeder and Green 2012; Donnelly and Green 2013; Murphy et al. 2018). These observations suggest that obesity impairs the rate of the secondary phase of vasodilation and hyperemia in contracting muscles.

The similar time delays between the second phase of the $\dot{V}$O_2_ response during cycling and second growth phase (phase 3) of the LVC response during calf exercise suggest that these phases represent the same general phase of an overall dynamic physiological response to exercise. The positive correlation between the time constants of these two phases, albeit not quite significant (*P* = 0.07), supports the prospect of a causal link between vasodilation and $\dot{V}$O_2_ dynamics. This, combined with the relatively larger effect of BMI on LVC dynamics (phase 3) compared with $\dot{V}$O_2_ dynamics raises the possibility that a slowing of vasodilation in contracting muscles contributes to the BMI‐related slowing of $\dot{V}$O_2_ dynamics observed during cycling.

### Becoming overweight

A question arises about the timing of effects of BMI on $\dot{V}$O_2_ and cardiovascular dynamics as subjects gain weight, and whether or not any of these effects appear ‘early’ in overweight subjects. Although $\dot{V}$O_2_ dynamics during cycling were not significantly slowed in overweight subjects, their BMI and *τ*_2_ ($\dot{V}$O_2_) values were intermediate and equidistant between lean and obese values. This suggests that there is a proportional effect of BMI on *τ*_2_ and that important effects might appear in overweight subjects.

With respect to LVC dynamics during calf exercise, the time delays of the second (decay) and third (growth) phase were greater in overweight subjects compared with lean subjects but similar between lean and obese subjects. These effects, however, were observed only in female subjects. These data raise the possibility that an increase in body mass and transition from lean to overweight categories results in changes to the timing of later phases of vascular responsiveness and which precede the slowing of vasodilation seen with larger increases in BMI, at least in females.

### Lipids and vasodilation

Correlations between several plasma lipid markers (total cholesterol, HDL, triglycerides) and *τ*_3_ of the LVC response, but not *τ*_2_ of the $\dot{V}$O_2_ response, are broadly consistent with the detrimental effects of some lipid species on vascular reactivity in human limbs (Steinberg et al. 1997) and skeletal muscle (Clerk et al. 2002; Steinberg and Baron 2002). These correlations raise the possibility that changes in blood lipids which accompany weight gain might affect vascular dynamics in contracting muscles and possibly contribute to the present findings. Further research is needed to clarify this.

### Limitations

Despite its limitations, BMI is recognized as a useful measurement of obesity (Ortega et al. 2016). Leg skinfold estimates (Results) along with muscle mass and volume measurements (Table 1) show that increasing BMI was associated with increasing skinfold values and a relatively smaller muscle mass per limb volume, measurements consistent with increasing adiposity. Whether the effects of BMI on $\dot{V}$O_2_ and cardiovascular dynamics can be attributed to physiological effects of obesity per se or related factors (e.g., age, blood pressure, inactivity) requires clarification. Nevertheless, with the exception of BMI and body mass, none of these other factors (see characteristics in Table 1) were correlated with BMI or parameters of $\dot{V}$O_2_ dynamics despite their wide and normal distributions. The use of males and females to explore effects of BMI might be criticized on the basis of observations of sex differences in $\dot{V}$O_2_ dynamics in overweight and obese individuals with type 2 diabetes (Regensteiner et al. 2015). However, these observations are not consistent with our findings (O'Connor et al. 2012) or a review of all studies of males and females with type 2 diabetes (Green et al. 2015). Moreover, the weak effects of sex (*F* < 1.0) on parameters describing $\dot{V}$O_2_ dynamics and most LVC variables supports our use of males and females in this study. We acknowledge that a larger and more even sample of males and females is required for studies of sex effects.

### Perspective

Obesity reduces cardiorespiratory fitness but it is not clear whether this effect is a function of increased *weight per se* on estimates of fitness or reflects direct effects underlying physiological function (see Introduction). Assessment of $\dot{V}$O_2_ dynamics during submaximal exercise that is not weight‐bearing, combined with the absence of normalization of parameter estimates to body mass, helps overcome the confounding problem of body mass. The speed with which $\dot{V}$O_2_ and underlying cardiorespiratory function adjusts to the onset of submaximal exercise is probably as important an indicator of cardiorespiratory ‘fitness’ as a peak or maximum exercise response. Present findings show that obesity impairs such dynamic responses and, given practical problems with the assessment of peak and maximum responses (Poole and Jones 2017; Green and Askew 2018), has implications for further study of effects of obesity on cardiorespiratory fitness.

## Conclusion

In conclusion, the present findings show that BMI is associated with a slowing of the dynamic responses of $\dot{V}$O_2_ (*τ*_2_) during cycling and leg vascular conductance during isolated limb exercise in apparently healthy adults of both sexes. Further study is required to verify these effects and clarify the mechanisms involved.

## Conflicts of Interest

All authors have no conflicts of interest to declare.
